# Supplementary material for: Perineuronal nets control visual input via thalamic recruitment of cortical PV interneurons
Source: eLife. 2018 Dec 18;7:e41520. doi: 10.7554/eLife.41520 (PMC6298774; doi:10.7554/eLife.41520)
Supplement: Supplementary file 1. [file elife-41520-supp1.docx]

Supplementary File 1

**Perineuronal nets control visual input via thalamic recruitment of cortical PV interneurons**

Giulia Faini^1^, Andrea Aguirre^1^, Silvia Landi^2^, Tommaso Pizzorusso^3,4^, Gian Michele Ratto^2^, Charlotte Deleuze^1^, Alberto Bacci^1^

Correspondence to: [alberto.bacci@icm-institute.org](mailto:alberto.bacci@icm-institute.org)

**Table S1: Passive properties of PV cells and PNs following PNN removal and monocular deprivation.**

| **PV cells** | n | N | First injected current* (pA) | Rm (MΩ) | Vrest (mV) |
| --- | --- | --- | --- | --- | --- |
| Sham | 26 | 12 | -115.27 ± 6.57 | 96.2 ± 6.2 | -66.31 ± 0.87 |
| ChABC | 25 | 9 | -104.68 ± 4.98 | 96.02 ± 4.5 | -65.47 ± 0.92 |
| Sham + MD | 10 | 5 | -75.40 ± 6.65 | 144.7 ± 13 | -63.67 ± 1.7 |
| ChABC + MD | 13 | 8 | -88.23 ± 7.99 | 121.6 ± 13 | -61.57 ± 1.4 |

| **PNs** | n | N | First injected current* (pA) | Rm (MΩ) | Vrest (mV) |
| --- | --- | --- | --- | --- | --- |
| Sham | 13 | 7 | -50.57 ± 3.04 | 248.7 ± 39.9 | -68.73 ± 1.98 |
| ChABC | 9 | 6 | -49.56 ± 6.64 | 252.5 ± 44.1 | -69.04 ± 3.3 |
| Sham + MD | 8 | 4 | -47.42 ± 6.24 | 261.4 ± 33 | -69.5 ± 1.9 |
| ChABC + MD | 9 | 6 | -48.33 ± 6.04 | 253 ± 31 | -70.89 ± 0.85 |

Mean values ± SEM. n = number of cells; N = number of mice.

*First injected current to induce a ΔV of -10 mV; incremental current steps in *f-i* plots were half this value.

**Table S2: Action potential parameters of PV cells and PNs following PNN removal and monocular deprivation.**

| **PV cells** | n | N | AP threshold (mV) | AP width (ms) | AP peak (mV) |
| --- | --- | --- | --- | --- | --- |
| Sham | 12 | 7 | -48.95 ± 1.10 | 0.32 ± 0.02 | 20.84 ± 1.5 |
| ChABC | 10 | 5 | -50.45 ± 0.75 | 0.31 ± 0.017 | 23.04 ± 1.6 |
| Sham + MD | 8 | 6 | -51.92 ± 0.92 | 0.39 ± 0.015 | 17.59 ± 1.8 |
| ChABC + MD | 10 | 6 | -51.71 ± 0.89 | 0.38 ± 0.014 | 18.58 ± 1.4 |

| **PNs** | n | N | AP threshold (mV) | AP width (ms) | AP peak (mV) |
| --- | --- | --- | --- | --- | --- |
| Sham | 12 | 9 | -47.98 ± 1.4 | 0.87 ± 0.07 | 29.49 ± 3.2 |
| ChABC | 9 | 4 | -49.18 ± 1.8 | 0.95 ± 0.03 | 28.40 ± 3.3 |
| Sham + MD | 8 | 5 | -50.56 ± 1.3 | 1.11 ± 0.08 | 28.66 ± 3.1 |
| ChABC + MD | 9 | 5 | -49.97 ± 1.3 | 1.03 ± 0.03 | 23.18 ± 2.3 |

Mean values ± SEM. n = number of cells; N = number of mice.

**Table S3: Glutamatergic neurotransmission onto PV cells and PNs following PNN degradation and monocular deprivation.**

| **PV cells** | n | N | sEPSC ampl. (pA) | sEPSC freq. (Hz) |
| --- | --- | --- | --- | --- |
| Sham | 29 | 14 | 9.81 ± 0.4 | 33.50 ± 3.0 |
| ChABC | 24 | 9 | 11.88 ± 0.5 | 46.80 ± 4.7 |
| Sham + MD | 14 | 6 | 9.52 ± 0.7 | 20.15 ± 2.5 |
| ChABC + MD | 23 | 9 | 9.46 ± 0.2 | 34.47 ± 2.2 |

| **PNs** | n | N | sEPSC ampl. (pA) | sEPSC freq. (Hz) |
| --- | --- | --- | --- | --- |
| Sham | 16 | 12 | 7.94 ± 0.4 | 7.70 ± 1.2 |
| ChABC | 15 | 9 | 8.80 ± 0.7 | 9.11 ± 1.3 |
| Sham + MD | 9 | 5 | 7.25 ± 0.7 | 5.43 ± 0.8 |
| ChABC + MD | 12 | 8 | 8.47 ± 0.5 | 7.80 ± 0.8 |

| **PV cells** | n | N | mEPSC ampl. (pA) | msEPSC freq. (Hz) |
| --- | --- | --- | --- | --- |
| Sham | 15 | 7 | 7.98 ± 0.4 | 27.30 ± 2.5 |
| ChABC | 13 | 8 | 7.65 ± 0.3 | 40.76 ± 2.9 |
| Sham + MD | 10 | 5 | 8.64 ± 0.4 | 22.34 ± 3.4 |
| ChABC + MD | 10 | 5 | 8.18 ± 0.4 | 22.43 ± 1.3 |

sEPSC: spontaneous excitatory postsynaptic currents. mEPSC: miniature excitatory postsynaptic currents recorded in TTX. Mean values ± SEM. n=number of cells, N=number of mice.

**Table S4: Thalamocortical glutamatergic responses on PV cells and PNs, in control conditions and after PNN removal and monocular deprivation.**

| **PV cells** | n | N | Area (pA*ms) | Light power (mW) | Fail. rate |
| --- | --- | --- | --- | --- | --- |
| Sham | 18 | 8 | 156 ± 22.7 | 0.26 ± 0.07 | 0.15 ± 0.09 |
| ChABC | 16 | 7 | 358.9 ± 85.5 | 0.14 ± 0.02 | 0.17 ± 0.04 |
| Sham + MD | 8 | 3 | 91.24 ± 15.0 | 0.18 ± 0.03 | 0.44 ± 0.03 |
| ChABC + MD | 8 | 3 | 169.7 ± 14.8 | 0.17 ± 0.03 | 0.49 ± 0.08 |

| **PNs** | n | N | Area (pA*ms) | Light power (mW) | Fail. rate |
| --- | --- | --- | --- | --- | --- |
| Sham | 8 | 4 | 146.1 ± 19.0 | 0.13 ± 0.02 | 0.35 ± 0.06 |
| ChABC | 8 | 3 | 162.7 ± 22.4 | 0.14 ± 0.01 | 0.41 ± 0.07 |
| Sham + MD | 8 | 4 | 95.85 ± 9.4 | 0.17 ± 0.03 | 0.36 ± 0.05 |
| ChABC + MD | 7 | 3 | 131.1 ± 27.4 | 0.18 ± 0.03 | 0.28 ± 0.07 |

Mean values ± SEM. n=number of cells, N=number of mice. Responses were measured at threshold in presence of TTX and 4-AP.

**Table S5:** **GABAergic neurotransmission onto PV cells and PNs following PNN degradation and MD.**

| **PV cells** | n | N | sIPSC ampl. (pA) | sIPSC freq. (Hz) |
| --- | --- | --- | --- | --- |
| Sham | 16 | 7 | 24.71 ± 2.0 | 7.33 ± 1.0 |
| ChABC | 17 | 7 | 37.60 ± 4.5 | 11.88 ± 1.7 |
| Sham + MD | 15 | 5 | 32.04 ± 2.6 | 4.44 ± 0.6 |
| ChABC + MD | 14 | 5 | 34.21 ± 3.4 | 7.03 ± 0.9 |

| **PNs** | n | N | sIPSC ampl. (pA) | sIPSC freq. (Hz) |
| --- | --- | --- | --- | --- |
| Sham | 11 | 6 | 40.33 ± 3.4 | 4.18 ± 0.51 |
| ChABC | 11 | 8 | 35.15 ± 3.6 | 5.49 ± 1.2 |
| Sham + MD | 14 | 4 | 30.83 ± 2.6 | 3.46 ± 0.8 |
| ChABC + MD | 13 | 4 | 34.22 ± 3.2 | 3.65 ± 0.5 |

| **PV cells** | n | N | mIPSC ampl. (pA) | mIPSC freq. (Hz) |
| --- | --- | --- | --- | --- |
| Sham | 12 | 5 | 35.13 ± 4.1 | 4.62 ± 0.6 |
| ChABC | 10 | 6 | 30.44 ± 3.6 | 5.14 ± 0.7 |
| Sham + MD | 10 | 3 | 35.88 ± 2.9 | 4.19 ± 0.4 |
| ChABC + MD | 8 | 3 | 35.81 ± 3.2 | 6.92 ± 1.4 |

sIPSCs: spontaneous inhibitory postsynaptic currents. mIPSCs: miniature inhibitory postsynaptic currents recorded in TTX. Mean values ± SEM. n=number of cells, N=number of mice.

**Table S6:** **Unitary GABAergic connections from PV cells, in control conditions and after PNN removal and monocular deprivation.**

| **PV-PV syn + aut** | n | N | Conductance (nS) |
| --- | --- | --- | --- |
| Sham | 33 | 10 | 5.146 ± 0.82 |
| ChABC | 26 | 11 | 5.550 ± 0.88 |
| Sham + MD | 11 | 4 | 3.268 ± 0.52 |
| ChABC + MD | 8 | 4 | 6.103 ± 1.61 |

| **PV-PN** | n | N | Conductance (nS) |
| --- | --- | --- | --- |
| Sham | 10 | 6 | 2.527 ± 0.7 |
| ChABC | 13 | 7 | 3.374 ± 0.9 |
| Sham + MD | 8 | 3 | 3.945 ± 1.3 |
| ChABC + MD | 9 | 5 | 5.672 ± 1.4 |

Mean values ± SEM. n=number of cells, N=number of mice.

**Table S7: Thalamocortical feed-forward inhibition on PV cells and PNs after PNN removal and monocular deprivation.**

| **PV cells** | n | N | Stim. | Amplitude  (pA) | Light power (mW) | Latency (ms) | Fail. rate |
| --- | --- | --- | --- | --- | --- | --- | --- |
| Sham | 10 | 7 | thresh | 208.4 ± 40.7 | 0.35 ± 0.05 | 7.13 ± 0.35 | 0.25 ± 0.03 |
| ChABC | 9 | 7 | thresh | 1095 ± 209 | 0.33 ± 0.06 | 6.09 ± 0.43 | 0.28 ± 0.07 |
| Sham + MD | 10 | 4 | thresh | 257.5 ± 41.2 | 0.34 ± 0.05 | 5.98 ± 0.26 | 0.37 ± 0.05 |
| ChABC + MD | 10 | 5 | thresh | 557.1 ± 122 | 0.30 ± 0.06 | 6.03 ± 0.19 | 0.33 ± 0.07 |

| **PNs** | n | N | Stim. | Amplitude (pA) | Light power (mW) | Latency (ms) | Fail. rate |
| --- | --- | --- | --- | --- | --- | --- | --- |
| Sham | 14 | 7 | thresh | 162.1 ± 19.32 | 0.35 ± 0.04 | 6.81 ± 0.29 | 0.42 ± 0.05 |
| ChABC | 16 | 6 | thresh | 195.2 ± 35.79 | 0.36 ± 0.05 | 6.59 ± 0.36 | 0.36 ± 0.05 |
| Sham + MD | 15 | 6 | thresh | 238.2 ± 32.12 | 0.33 ± 0.03 | 6.11 ± 0.15 | 0.39 ± 0.03 |
| ChABC + MD | 14 | 5 | thresh | 404 ± 75.96 | 0.24 ± 0.03 | 6.17 ± 0.16 | 0.32 ± 0.03 |
| Sham | 7 | 2 | x1.5 | 860.4 ± 156 | 0.35 ± 0.04 | 5.2 ± 0.21 | / |
| ChABC | 7 | 2 | x1.5 | 1715 ± 255 | 0.28 ± 0.02 | 5.1 ± 0.16 | / |
| Sham + MD | 11 | 4 | x1.5 | 1081 ± 172 | 0.39 ± 0.04 | 5.1 ± 0.09 | / |
| ChABC + MD | 11 | 4 | x1.5 | 1257 ± 199 | 0.39 ± 0.04 | 5.2 ± 0.11 | / |

Mean values ± SEM. n=number of cells, N=number of mice. Responses were measured at threshold stimulation (thresh.) or at 1.5 x thresh. (x1.5).

**Table S8: Glutamatergic and GABAergic neurotransmission onto PV cells and PNs during development.**

| **PV cells** | n | N | sEPSC ampl. (pA) | sEPSC freq. (Hz) |
| --- | --- | --- | --- | --- |
| < P20 | 16 | 9 | 15.79 ± 0.5 | 27.85 ± 1.6 |
| P25-P32 | 18 | 7 | 13.53 ± 0.4 | 37.40 ± 3.9 |
| P40-P60 | 22 | 11 | 11.47 ± 0.6 | 36.73 ± 3.4 |
| > P70 | 27 | 9 | 11.27 ± 0.41 | 25.40 ± 1.9 |
|  |  |  | sIPSC ampl. (pA) | sIPSC freq. (Hz) |
| < P20 | 12 | 5 | 32.24 ± 3.2 | 7.29 ± 1.7 |
| P25-P32 | 9 | 4 | 26.00 ± 1.6 | 6.67 ± 1.0 |
| P40-P60 | 10 | 6 | 25.01 ± 2.9 | 7.56 ± 1.3 |
| > P70 | 10 | 5 | 30.65 ± 3.4 | 6.35 ± 0.9 |

| **PNs** | n | N | sEPSC ampl. (pA) | sEPSC freq. (Hz) |
| --- | --- | --- | --- | --- |
| < P20 | 11 | 5 | 7.37 ± 0.4 | 8.03 ± 1.4 |
| P25-P32 | 12 | 6 | 9.53 ± 0.6 | 7.24 ± 0.9 |
| P40-P60 | 18 | 7 | 8.28 ± 0.6 | 8.66 ± 1.4 |
| > P70 | 13 | 6 | 9.55 ± 0.7 | 5.82 ± 1.2 |
|  |  |  | sIPSC ampl. (pA) | sIPSC freq. (Hz) |
| < P20 | 10 | 2 | 36.26 ± 3.5 | 3.013 ± 0.6 |
| P25-P32 | 7 | 3 | 35.01 ± 3.4 | 5.68 ± 1.0 |
| P40-P60 | 10 | 5 | 34.47 ± 3.3 | 4.60 ± 0.6 |
| > P70 | 9 | 4 | 36.50 ± 4.5 | 5.91 ± 1.1 |

sEPSC: spontaneous excitatory postsynaptic currents. sIPSC: spontaneous inhibitory postsynaptic currents. Mean values ± SEM. n=number of cells, N=number of mice.

`
