# Supplementary material for: Perineuronal nets control visual input via thalamic recruitment of cortical PV interneurons
Source: eLife. 2018 Dec 18;7:e41520. doi: 10.7554/eLife.41520 (PMC6298774; doi:10.7554/eLife.41520)
Supplement: Transparent reporting form [file elife-41520-transrepform.pdf]

eLIFE
1st Floor
24 Hills Road
Cambridge CB2 1JP, UK
P 01223 855340
W elifesciences.org
T @elife

eLife's transparent reporting form

We encourage authors to provide detailed information *within their submission* to facilitate the interpretation and replication of experiments. Authors can upload supporting documentation to indicate the use of appropriate reporting guidelines for health-related research (see EQUATOR Network), life science research (see the BioSharing Information Resource), or the ARRIVE guidelines for reporting work involving animal research. Where applicable, authors should refer to any relevant reporting standards documents in this form.

If you have any questions, please consult our Journal Policies and/or contact us: editorial@elifesciences.org.

Sample-size estimation

- You should state whether an appropriate sample size was computed when the study was being designed
- You should state the statistical method of sample size computation and any required assumptions
- If no explicit power analysis was used, you should describe how you decided what sample (replicate) size (number) to use

Please outline where this information can be found within the submission (e.g., sections or figure legends), or explain why this information doesn't apply to your submission:

No sample-size calculation was performed. All data were subject to statistical tests to decide whether parametric or nonparametric tests should be applied. Sample sizes were chosen depending on the type of experiment and the difficulty of obtaining good-quality recordings. Recordings of spontaneous synaptic activity were done with the highest possible samples (number of recorded neurons and mice), due to intrinsic variability. For optogenetic experiments, requiring multiple surgeries, the sample size was inherently lower, due to technical constraints. In any case, the sample size was never smaller than 7 in all experiments. Sample size was comparable to other published studies using similar approaches.

No data passing a quality control was excluded from the analysis. Recorded neurons were excluded from the analysis when series resistance exceeded 30 MOhm. No data was excluded from the analysis a posteriori.

All this is indicated in the Materials and Methods section of the paper.

Replicates

- You should report how often each experiment was performed
- You should include a definition of biological versus technical replication
- The data obtained should be provided and sufficient information should be provided to indicate the number of independent biological and/or technical replicates
- If you encountered any outliers, you should describe how these were handled
- Criteria for exclusion/inclusion of data should be clearly stated

eLife Sciences Publications, Ltd is a limited liability non-profit non-stock corporation incorporated in the State of Delaware, USA, with company number 5030732, and is registered in the UK with company number FC030576 and branch number BR015634 at the address 1st Floor, 24 Hills Road, Cambridge CB2 1JP | August 2014

- High-throughput sequence data should be uploaded before submission, with a private link for reviewers provided (these are available from both GEO and ArrayExpress)

Please outline where this information can be found within the submission (e.g., sections or figure legends), or explain why this information doesn't apply to your submission:

Replication was ensured by repeating the experiments several times, interleaving different age groups and/or experimental conditions and treatments. In our hands, experimental protocols and results were reliably reproduced as it could be judged from the figures and associated statistics. The number of neurons recorded (n) and number of mice used (N) are indicated in the Results sections and in the supplementary tables. Summary plots of Figs. 2-5 illustrate results obtained from single neurons and corresponding population data. This allows visual control of variability, outliers and the actual number of neurons recorded.

eLife Sciences Publications, Ltd is a limited liability non-profit non-stock corporation incorporated in the State of Delaware, USA, with company number 5030732, and is registered in the UK with company number FC030576 and branch number BR015634 at the address 1st Floor, 24 Hills Road, Cambridge CB2 1JP | August 2014

Statistical reporting

- Statistical analysis methods should be described and justified
- Raw data should be presented in figures whenever informative to do so (typically when N per group is less than 10)
- For each experiment, you should identify the statistical tests used, exact values of N, definitions of center, methods of multiple test correction, and dispersion and precision measures (e.g., mean, median, SD, SEM, confidence intervals; and, for the major substantive results, a measure of effect size (e.g., Pearson's r, Cohen's d)
- Report exact p-values wherever possible alongside the summary statistics and 95% confidence intervals. These should be reported for all key questions and not only when the p-value is less than 0.05.

Please outline where this information can be found within the submission (e.g., sections or figure legends), or explain why this information doesn't apply to your submission:

Statistical analyses and tests used are explained in the 'Statistical Tests' subsection of the Methods section. All data were subject to statistical tests to decide whether parametric or nonparametric tests should be applied. Raw data are presented for all figures.

(For large datasets, or papers with a very large number of statistical tests, you may upload a single table file with tests, Ns, etc., with reference to sections in the manuscript.)

Group allocation

- Indicate how samples were allocated into experimental groups (in the case of clinical studies, please specify allocation to treatment method); if randomization was used, please also state if restricted randomization was applied
- Indicate if masking was used during group allocation, data collection and/or data analysis

Please outline where this information can be found within the submission (e.g., sections or figure legends), or explain why this information doesn't apply to your submission:

Different treatments (ChABC vs. sham) or experimental conditions (MD vs. control) or age groups were allocated randomly across mice. This is stated in the 'Statistical tests' sub-section of the Methods section

Additional data files ("source data")

- We encourage you to upload relevant additional data files, such as numerical data that are represented as a graph in a figure, or as a summary table
- Where provided, these should be in the most useful format, and they can be uploaded as "Source data" files linked to a main figure or table
- Include model definition files including the full list of parameters used
- Include code used for data analysis (e.g., R, MatLab)
- Avoid stating that data files are "available upon request"

Please indicate the figures or tables for which source data files have been provided:

At the moment of submission, no original source data is provided because the first author is traveling and thus unavailable to provide source data files. We will be happy to provide all source data in the event the paper is accepted for publication and/or at any moment during the review of the manuscript.
